# Multi-scale *in silico* and *ex silico* mechanics of 3D printed cochlear implants for local drug delivery

**DOI:** 10.3389/fbioe.2023.1289299

**Published:** 2024-01-31

**Authors:** A. Isaakidou, M. Ganjian, R. van Hoften, M. C. Saldivar, M. A. Leeflang, A. Groetsch, M. Wątroba, J. Schwiedrzik, M. J. Mirzaali, I. Apachitei, L. E. Fratila-Apachitei, A. A. Zadpoor

**Affiliations:** ^1^ Department of Biomechanical Engineering, Faculty of Mechanical Engineering, Delft University of Technology (TU Delft), Delft, Netherlands; ^2^ Empa, Swiss Federal Laboratories for Materials Science and Technology, Laboratory of Mechanics of Materials and Nanostructures, Thun, Switzerland; ^3^ Department of Materials Science and Engineering, Henry Samueli School of Engineering, University of California, Irvine, Irvine, CA, United States

**Keywords:** 3D printing, two-photon polymerization, stereolithography, Raman spectroscopy, cochlear implant, finite element analysis, mechanical characterization, compression

## Abstract

The currently available treatments for inner ear disorders often involve systemic drug administration, leading to suboptimal drug concentrations and side effects. Cochlear implants offer a potential solution by providing localized and sustained drug delivery to the cochlea. While the mechanical characterization of both the implants and their constituent material is crucial to ensure functional performance and structural integrity during implantation, this aspect has been mostly overlooked. This study proposes a novel methodology for the mechanical characterization of our recently developed cochlear implant design, namely, rectangular and cylindrical, fabricated using two-photon polymerization (2 PP) with a novel photosensitive resin (IP-Q™). We used *in silico* computational models and *ex silico* experiments to study the mechanics of our newly designed implants when subjected to torsion mimicking the foreseeable implantation procedure. Torsion testing on the actual-sized implants was not feasible due to their small size (0.6 × 0.6 × 2.4 mm³). Therefore, scaled-up rectangular cochlear implants (5 × 5 × 20 mm³, 10 × 10 × 40 mm³, and 20 × 20 × 80 mm³) were fabricated using stereolithography and subjected to torsion testing. Finite element analysis (FEA) accurately represented the linear behavior observed in the torsion experiments. We then used the validated Finite element analysis models to study the mechanical behavior of real-sized implants fabricated from the IP-Q resin. Mechanical characterization of both implant designs, with different inner porous structures (pore size: 20 μm and 60 μm) and a hollow version, revealed that the cylindrical implants exhibited approximately three times higher stiffness and mechanical strength as compared to the rectangular ones. The influence of the pore sizes on the mechanical behavior of these implant designs was found to be small. Based on these findings, the cylindrical design, regardless of the pore size, is recommended for further research and development efforts.

## 1 Introduction

**FIGURE 1:**
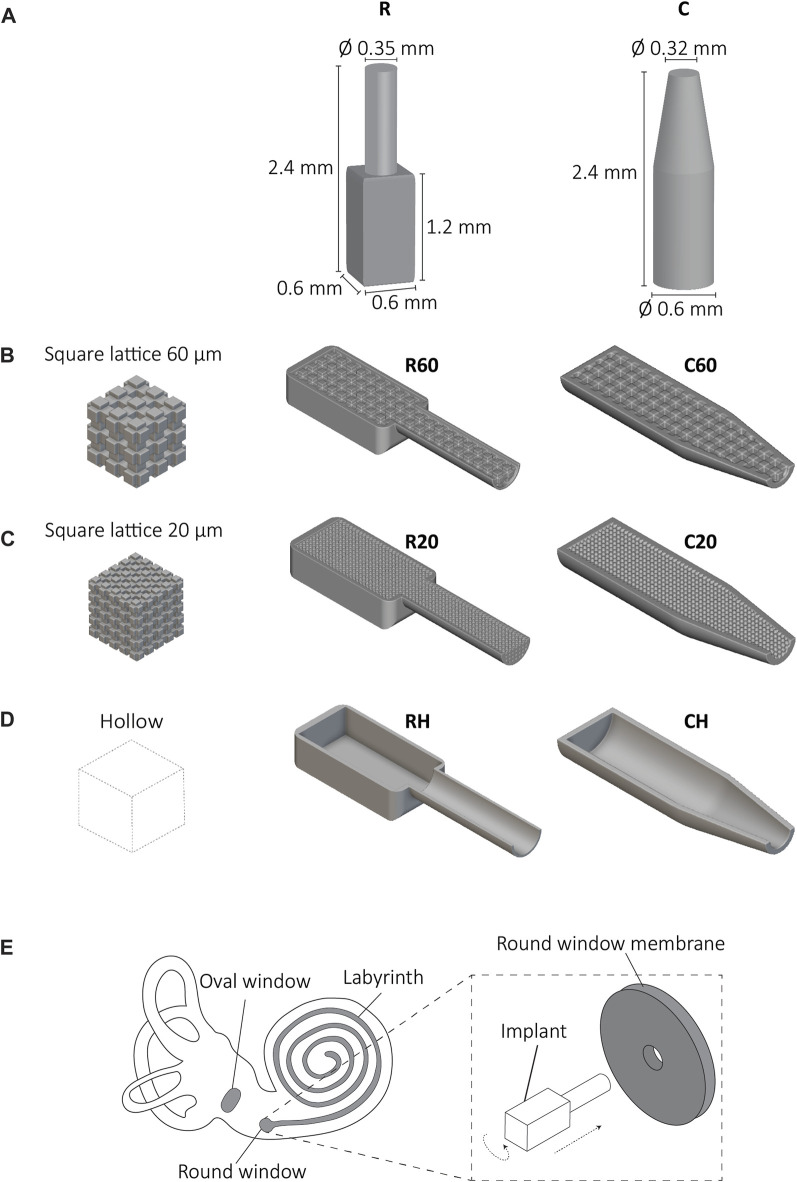
The cochlear implants and implantation method. **(A)** The cochlear implant designs with dimensions; rectangular (R) and cylindrical (C), The lateral views of the R and C implants with inner porous cubic lattice structures with a unit cell of **(B)** 60 × 60 × 60 μm^3^, **(C)** 20 × 20 × 20 μm^3^ and **(D)** hollow implants, **(E)** A schematic representation of the anatomy of the inner ear (*i.e.*, cochlea) including the labyrinth, the oval, and round window. The details of the proposed implantation method of the implant to the round window involve a translational and a rotational move.

The effective delivery of drugs to the inner ear, encompassing the vestibule and cochlea, is impeded by the presence of the blood-labyrinth barrier (BLB) (Zhang et al., 2021). Although essential for maintaining homeostasis and regulating ion and nutrient transportation, the BLB restricts the entry of high molecular weight compounds, such as drugs, hindering the treatment of inner ear disorders (Hao and Li, 2019; Szeto et al., 2020). Local drug delivery methods, such as microneedles, intratympanic injections, nanoparticles, stents, silicone-based rods, and implants offer a promising approach to enhance drug bioavailability within the target organ while minimizing the systemic side effects (*e.g.*, fatigue, nausea, headache, cardiovascular complications) associated with general drug administration (Hao and Li, 2019). Cochlear implants, with anatomically relevant sizes for the human ear (*i.e.*, 0.6 × 0.6 × 2.4 mm³), incorporating a drug reservoir and an implantable tip, have been recently developed as an alternative solution to the existing drug delivery methods by using two-photon polymerization (2PP) (Isaakidou et al., 2023). The implant shapes were rectangular (R) and cylindrical (C) featuring cylindrical tips (Figure 1A). To enable drug storage and release, these implants feature either an internal interconnected network of square pores in two different sizes (20 and 60 μm) (Figures 1B,C) or a hollow design (Figure 1D). As a result, six distinct implant designs were created, namely, R20, R60, RH, C20, C60, and CH (Figures 1B–D). The internal porosity fulfills a dual function: firstly, it acts as a reservoir for a particular pharmaceutical agent or drug associated with the auditory impairment, and secondly, it serves as a mechanism to modulate the precise release of the medication into the cochlear labyrinth. Furthermore, the architectural configuration of the implant, in conjunction with its porous characteristics, plays a vital role in preserving its structural stability in the phases preceding, throughout, and subsequent to the implantation process. The implant size and shape are crucial to fulfilling the anatomical requirements of the cochlea at the implantation site. These implants can be inserted through either the round window or the oval window of the cochlea, requiring twisting and insertion into a surgically created aperture (Figure 1E). As these implants undergo mechanical stresses during implantation, it is crucial to determine their mechanical properties to ensure the safety of the surgical procedure. However, due to their small size, complex geometry, and specific fabrication technique, conventional mechanical testing methods could not easily be used for comprehensive mechanical characterization of such implants. Utilizing the 2PP printing method is advantageous in producing small-scale structures with high precision. However, this method presents challenges when it comes to conducting microscale mechanical testing due to issues associated with implant-substrate adhesion. Microscale mechanical testing with commercially available micromechanical testing systems has been demonstrated feasible when the samples (micropillars, cantilevers, or dogbones) were prepared using dual beam systems (focused ion beam/scanning electron microscopy (FIB/SEM)) (Zhao et al., 2015; Jun et al., 2016; Ast et al., 2017; Fu et al., 2018). Finite element analysis (FEA) offers a numerical approach to simulate the mechanical behavior of complex structures and materials and has been employed for the modeling of compression testing of 2 PP printed origami architected metamaterials (Lin et al., 2020). In this regard, FEA can be used to simulate the mechanical behavior of cochlear implants during implantation, while experimental validation can be achieved using enlarged implant models. To the best of our knowledge, our study represents the first attempt to investigate the mechanical behavior of 3D-printed cochlear implants designed for local drug delivery. Thus, this study aimed to mechanically characterize the cochlear implants by first employing *in silico* FEA models for enlarged cochlear implants to investigate their mechanical behavior under compression and torsion. The results were then validated against *ex silico* experimental data obtained with enlarged implants fabricated using stereolithography (SLA) from Grey resin. The validated FEA models were finally used to help us assess the mechanical properties of real-size cochlear implants that are printed using 2PP from the IP-Q resin. Last, the results enabled us to choose the most potent design for further studies.

## 2 Materials and methods

### 2.1 Fabrication of the specimens for compression testing

#### 2.1.1 Printed pillars produced by two-photon polymerization (2PP)

Micro-pillars were printed using a Photonic Professional GT 2PP machine (Nanoscribe, Eggenstein-Leopoldshafen, Germany). The pillar geometry with a diameter of 50 µm and a height of 100 µm was designed using SolidWorks (Dassault Systèmes SE, France), exported as an STL (stereolithography) file and prepared for microscale printing using a Nanoscribe provided software Describe (Nanoscribe, Eggenstein-Leopoldshafen, Germany), in which the following printing parameters were set: laser power of 50 mW, scanning speed of 100,000 μm/s, hatching distance of 1 μm, and slicing distance of 5 µm. Samples were prepared by putting a droplet of IP-Q™ resin (Nanoscribe, Eggenstein-Leopoldshafen, Germany) onto a silicon substrate, which was then transferred to the Nanoscribe machine. For the two-photon absorption process, a femtosecond infrared laser beam with a wavelength of 780 nm was directed onto the resin. After the printing process, the fabricated specimen was immersed in propylene glycol monomethyl ether acetate (PGMEA, Sigma-Aldrich, Hamburg, Germany) for 25 min for development (removal of non-polymerized parts). Subsequently, it was rinsed in isopropyl alcohol (Sigma-Aldrich, Hamburg, Germany) for 5 min to remove any residual impurities. Finally, the specimen was dried thoroughly using an air-blowing gun. The dimensions of the printed pillars were measured using a high-resolution scanning electron microscope (SEM) (FEI Helios G4 CX dual-beam workstation, Hillsboro, OR, United States of America). To counteract charging for the polymeric samples, the specimens were gold-sputtered using a sputter coater (JFC-1300, JEOL, Akishima, Japan) for 40 s prior to SEM imaging.

#### 2.1.2 Printing of larger pillars by stereolithography (SLA)

Pillars were printed in triplicate (*n* = 3) using a Form 3+ SLA printer (Formlabs Inc., Berlin, Germany) The pillar geometry with a diameter of 8 mm and a height of 16 mm was created using SolidWorks (Dassault Systèmes SE, Vélizy-Villacoublay, France), exported as an STL file and was then imported into the preparation software of the 3D printer (PreForm 3.24.2, Formlabs Inc., Berlin, Germany), which allowed adjustments to be made for size, structure orientation, and layer thickness. For the printing process, Grey resin (Formlabs Inc., Berlin, Germany) was selected as the material due to its elastic properties, which were expected to closely resemble those of IP-Q. Two different printing orientations were employed, namely, horizontal and vertical, to investigate their effects on the resulting structures. Additionally, two different layer thicknesses, namely, 25 μm and 50 μm, were utilized to examine their impact on the printed pillars. Upon completion of the printing process, the build platform, containing the printed structures, was carefully removed from the printer. It was then immersed in isopropyl alcohol (IPA) for 10 min to dissolve any residual materials. Subsequently, the printed structures underwent post-curing under UV light at 80°C for 15 min, following the manufacturer’s instructions. The final geometry of the printed pillars, including height and diameter, were measured using a caliper with a precision of 0.05 mm. These measurements allowed for the evaluation and characterization of the printed pillar dimensions.

### 2.2 Fabrication of enlarged 3D printed implants for torsion testing

**FIGURE 2:**
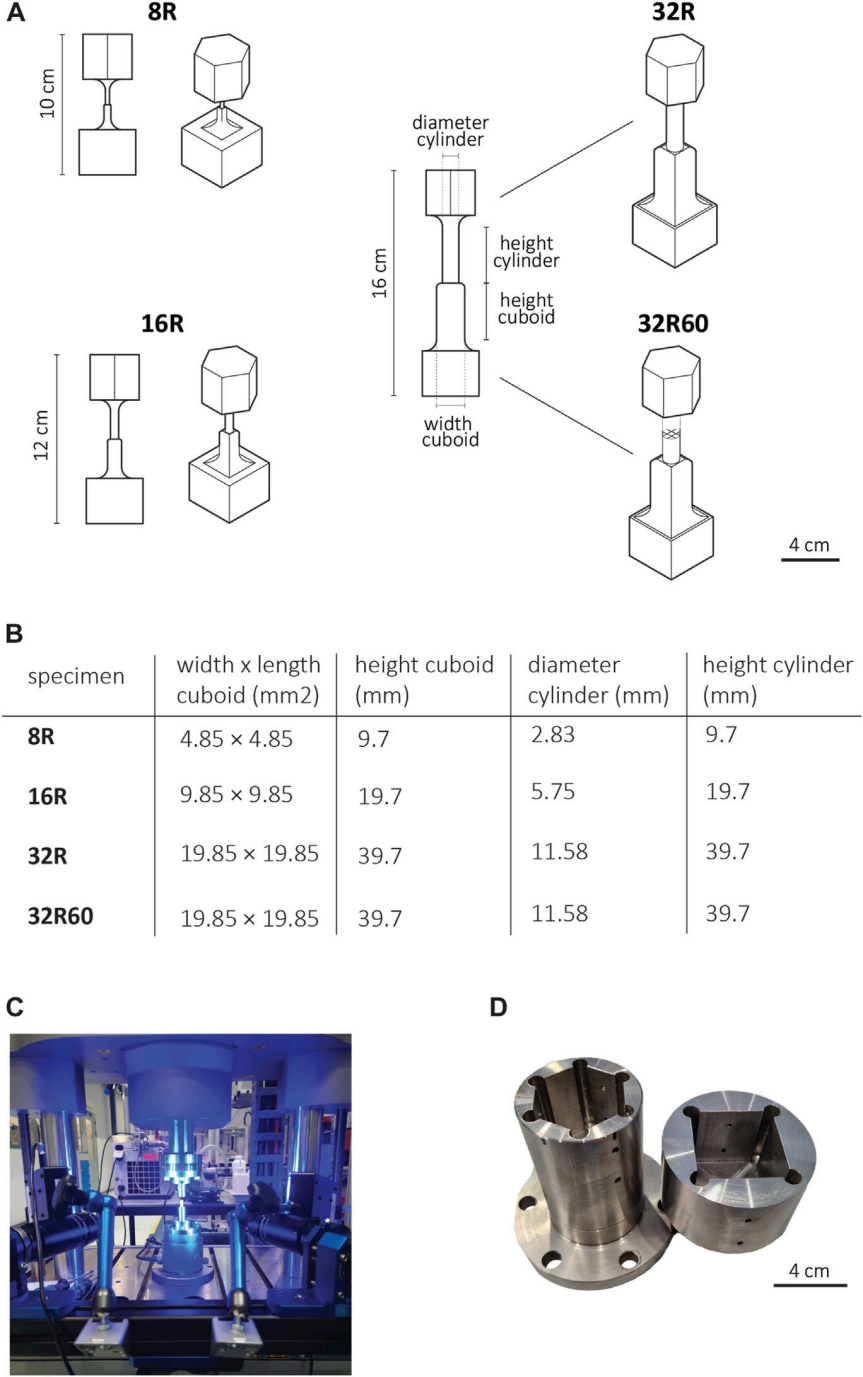
**(A)** The scaled-up R implant designs, including prismatic grippers (top: hexagonal, bottom: square) for torsion testing, **(B)** The design parameters of the scaled-up R implants for both the solid (8R, 16R, 32R) and porous (32R60) versions. **(C)** An image of the mechanical testing setup including Electropulse 10,000 and the DIC system. **(D)** The steel holders with prismatic sockets (top: hexagonal, bottom: square) used in torsion testing.

In this study, upscaled models of recently developed porous cochlear implant designs (Isaakidou et al., 2023) were modified and fabricated specifically for torsion testing. Four different specimen types were developed, namely, 8R, 16R, 32R, and 32R60, where the letter ‘R’ (rectangular) represents the implant type, the number preceding it (8, 16, or 32) corresponds to the scaling factor relative to the actual implant size, and the subsequent number after the letter R (if present) indicates the pore size (in μm) in the original porous design (Figure 2A).

To facilitate torsion testing, additional grippers were integrated into both ends of the implants. These grippers were designed using SolidWorks (Dassault Systèmes SE, Vélizy-Villacoublay, France) and were consistent in size across all three upscaled models. The upper gripper, located near the cylindrical part of the implant, was designed as a hexagonal prism with a base edge length of 19.93 mm and a height of 31.88 mm. The lower gripper, situated close to the cuboid part of the implant, was designed as a square prism with the dimensions of 39.85 mm × 39.85 mm × 31.88 mm (*w* × *h* × *l*) (Figures 2B,C). To minimize stress concentrations at the joints, additional fillets with a radius of 8 mm were incorporated into the design, ensuring a smoother transition from the implant to the grippers.

The models were fabricated in triplicate (*n* = 3) using the aforementioned SLA system (Form 3B+, Formlabs Inc., Berlin, Germany) with a layer thickness of 50 μm vertically oriented on the built plate. Grey resin was utilized as the printing material, following the methodology described earlier. The resulting geometry of the printed torsion specimens was measured using a caliper with a precision of 0.05 mm (Table 1).

**TABLE 1:** The dimensions of the torsion specimens 3D printed from Grey resin as measured by a caliper with a precision of 0.05 mm.

Specimen	Width cuboid (mm)	Diameter of cylinder (mm)	Height (mm)
8R_1	5.00	3.00	35.55
8R_2	5.00	2.95	35.15
8R_3	5.00	3.00	35.25
16R_1	10.00	5.85	55.00
16R_2	10.05	5.90	54.85
16R_3	9.95	5.90	55.10
32R_1	20.00	11.70	95.00
32R_2	20.05	11.75	94.90
32R_3	20.10	11.75	94.90
32R60_1	20.35	11.45	84.80
32R60_2	19.85	11.40	85.30
32R60_3	19.90	11.45	85.20

### 2.3 *In silico* investigation of torsion specimens and cochlear implants

FEA simulations were performed using Abaqus Standard (Dassault Systèmes Simulia, Vélizy-Villacoublay, France). The torsion testing was modeled for both the enlarged and real-sized implants. Variations with and without grippers as well as solid and porous designs were simulated for the enlarged specimens while variations of solid, hollow, and porous designs with pore sizes of 20 and 60 μm were simulated for the real-sized implants. The computational models of the torsion specimens required such inputs as the Young’s modulus, Poisson’s ratio, true stress, and true plastic strain. The Young’s modulus and Poisson’s ratio were derived from the compression tests on the Grey resin pillars, while the true stress and true plastic strain were calculated using Eq. 1 and Eq. 2), respectively. The outputs of the computational models included the forces (torque) and displacements of the structures, which were then converted into true stresses and strains. To assess the accuracy of the models, a comparison was made between the FEA results and the experimental data. The simulation of torsion testing for the R and C types of implants (solid, hollow, and porous) involved a pre-processing step that transformed the STL files into hexahedral element meshes, following the methodology developed and described by Saldivar et al. (Saldívar et al., 2022). The results of the mesh sensitivity analysis are provided in Supplementary Materials 1.1 and Supplementary Figure S1. This analysis showed that the stress results converged to less than 1% difference when the element number reached 25,000. The torque, rotation, shear stress, and shear strain were extracted for analysis.

### 2.4 *Ex silico* mechanical characterization

#### 2.4.1 Compression of the IP-Q micropillars

The IP-Q micropillars, with a diameter of 50 μm and a height of 100 μm, were subjected to compression using a commercial *ex situ* indenter setup (Alemnis AG, Thun, Switzerland) equipped with a flat punch diamond tip of 50 μm diameter. Micropillars were compressed uniaxially with a displacement-controlled loading protocol up to a maximum displacement of 30 μm at a quasi-static strain rate (1 × 10^−3^ s^-1^). To determine the engineering stress (σ_eng_), the top cross-sectional area of the pillars was used, while the engineering strain (ε_eng_) was calculated based on the initial height of the pillars (measured by SEM). The Young’s modulus was obtained by analyzing the slope in the initial elastic region, which corresponded to an engineering strain range of 10%–15%.

#### 2.4.2 Compression and torsion of the grey resin printed specimens

Compression and torsion testing of the SLA printed specimens was conducted using a dynamical testing machine (ElectropulseTM E10000, Instron Systems, Norwood, United States) (Figure 2C). The compression tests were performed at a strain rate of 1.3 mm/min, following the ASTM D695-15 standard (ASTM International, 2019), with a maximum loading of 8500 N. To study the full-field strain distributions, the local displacement fields of each test were captured using a Q-400 2 × 12 MPixel digital image correlation (DIC) system (LIMESS GmbH, Krefeld, Germany) at a frequency of 1 Hz. Before testing, a black dot speckle pattern was applied over a white paint background on each specimen. The strains of the tested specimens were determined using the commercial DIC program Instra 4D v4.6 (Danted Dynamics A/S, Skovunde, Denmark). The elastic modulus was calculated from the engineering stress-strain curve within the elastic region (0.3%–2% strain) using MATLAB R2020b, and the average value was obtained for each set of three specimens. The yield strength (*σ_y_*) was calculated at 0.2% strain. The true stress and strain were calculated from the engineering stress and strain using Eq. (1) and (2) (Arasaratnam et al., 2011):

$$\varepsilon =\ln\left(1+\right.\varepsilon_{eng}\;)\;$$ (1)

$$\sigma =\sigma_{eng}\left(1+\varepsilon_{eng}\right)$$ (2)

where *ε* is the true strain and *σ* is the true stress. The true plastic strain of the material was calculated using Eq. (3) (12):

εpl=εt− εel=εt− σΕ (3)

where *ε_pl_* is the true plastic strain, *ε_t_* is the true total strain, *ε_el_* is the true elastic strain, and Ε is the Young’s modulus.

For torsion testing, special steel holders were required, which were designed in SolidWorks and manufactured using a 5-axis computer numerical control (CNC) machine (MillTap 700, DMG MORI, Veenendaal, NL) (Figure 2D).

The torsion specimens were rotated at a rate of 0.001 s^-1^ according to ASTM E143-20. The rotational speed was adjusted to the specimen size (Table 2) using Eq. (4) (ASTM International, 2020):

γ=θrL (4)

where *γ* is the torsional shear strain, *θ* is the angle of rotation, r is the radius of the specimen, and *L* is the total length of the specimen.

**TABLE 2:** The rotational speed and strain rate for the solid and porous torsion specimens.

Specimen (R)	Torsional shear strain rate (s^-1^)	Rotational speed (rad/s)
8R	0.001	0.0238
16R		0.0183
32R		0.0158
32R60		0.0149

The torque (*T*) and rotation angle (*θ*) were recorded until failure or until reaching the torque limit (95 Nm) or rotation angle limit (270°). The DIC system was utilized to calculate the torsional shear strain for improved measurement accuracy. The torsional shear stress was determined from the recorded torque using Eq. (5) (ASTM International, 2020):

τ=TrJ (5)

where *τ* is the shear stress and *J* is the second polar moment of area. The polar moment of the area was calculated for the smallest cross-sectional area (*i.e.*, the cylindrical tip of the R implant) according to Eq. (6) (ASTM International, 2020):

J=πD432 (6)

where *D* is the diameter of the cylindrical tip of the torsion specimens (Table 1).

The strain was calculated by utilizing the DIC displacement values for two points on the cylindrical part of the specimens, near the base and the top. The recorded displacements were converted to rotational displacements using coordinate system conversion for cylindrical coordinates. The shear strain was then calculated using Eq. 4 and the shear modulus was calculated according to Eq. (7):

$$E=2G\left(1+v\right)$$ (7)

where G is the shear modulus and v is the Poisson’s ratio.

### 2.5 Fractography

To investigate the failure mode of the specimens after the torsion test, high-resolution SEM images of 8R specimens were captured from the fracture surface using a Helios microscope (FEI Helios G4 CX dual-beam workstation, Hillsboro, OR, United States) operating at 10 kV and 25 pA. Before SEM imaging, the specimens were coated with a thin layer of gold (thickness ≈5 nm) using a sputter coater (JFC-1300, JEOL, Japan). Optical images were also taken from the section closest to the cuboidal end of the torsion specimens following the test, and whenever possible, the fracture angles were determined.

### 2.6 Raman spectroscopy

The chemistry of uncured and cured IP-Q and Grey resin was analyzed using an inVia Reflex Raman system (Renishaw plc, Wotton-under-Edge, United Kingdom). Raman spectra were acquired using a ×50 objective lens, operated at an excitation wavelength of 532 nm. The laser intensity was set to 50%. Each spectrum was obtained by averaging 15 acquisitions with an exposure time of 1.0 s. Subsequently, a baseline correction was applied to subtract instrument noise using OriginPro 9.8.0.200 (OriginLab Corporation, Northampton, United States).

### 2.7 Statistical analysis

To investigate whether the Young’s modulus and yield strength of the Grey resin micropillars were significantly different with respect to the printing layer thickness and orientation, we conducted a one-way ANOVA test (*p* ≤ 0.05). We have assessed the normality of the data using the Shapiro-Wilk test (*p* ≤ 0.05), which is appropriate for the small sample size in our study. The results of these normality tests for the elastic moduli and compressive strength of the vertically and horizontally printed specimens are presented in Supplementary Figure S4. The analyses were performed using Prism 10 (GraphPad Software Inc., San Diego, CA, United States).

## 3 Results

### 3.1 Compression of the IP-Q (2PP) and grey resin (SLA) pillars

**FIGURE 3:**
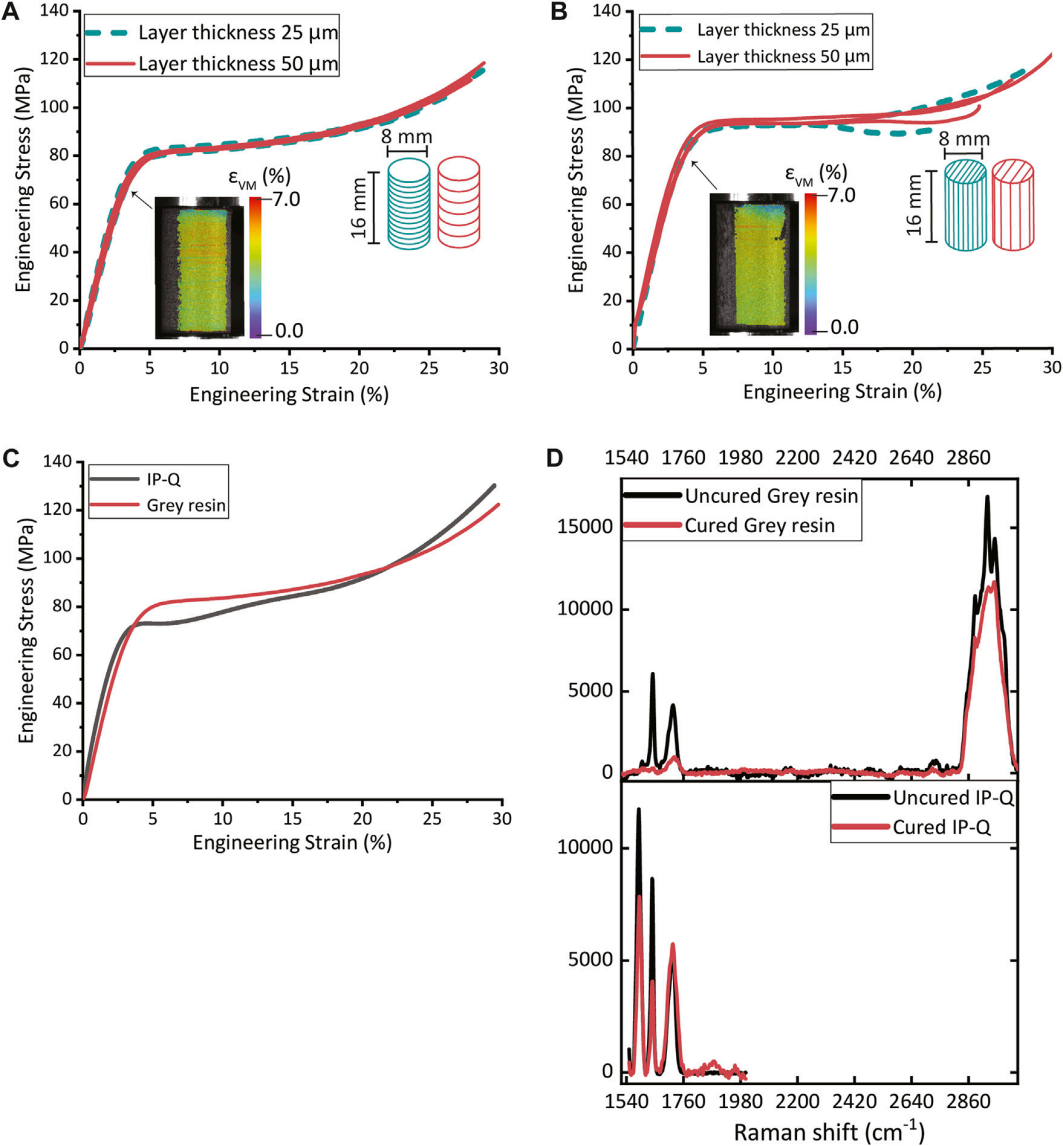
The compression stress-strain curves of **(A)** Grey resin pillars (ø 8 mm × 16 mm) with horizontally printed layers with a thickness of 25 μm or 50 μm (inset: DIC image of a compressed pillar at yield). **(B)** Grey resin pillars (ø 8 mm × 16 mm) with vertically printed layers with a thickness of 25 μm or 50 μm (inset: DIC image of a compressed pillar at yield). **(C)** The compression stress-strain curves for pillars printed from Grey resin and IP-Q. **(D)** The Raman spectra of polymerized (red) and unpolymerized (black) Grey resin and IP-Q specimens.

Micropillar compression tests revealed a Young’s modulus of 2.78 GPa and a yield strength of 60.4 MPa for the IP-Q printed pillars (Supplementary Figure S2). Compression testing was employed to calculate the Young’s modulus and yield strength of the pillars printed with two different layer thicknesses and in two different printing directions. The vertically printed pillars with a layer thickness of 25 µm exhibited a Young’s modulus of 2.51 ± 0.13 GPa and a yield strength of 59.2 ± 0.91 MPa, while those with a layer thickness of 50 µm had a Young’s modulus of 2.50 ± 0.10 GPa and a yield strength of 56.4 ± 1.7 MPa. The horizontally printed pillars displayed a Young’s modulus and yield strength of 2.51 ± 0.05 GPa and 72.4 ± 3.4 MPa for the 25 µm pillars, and 2.33 ± 0.20 GPa and 79.4 ± 2.8 MPa for the 50 µm pillars. The stress-strain curves obtained for the pillars with varying layer thicknesses and printing orientations revealed that there were no statistically significant differences in the Young’s modulus values. However, statistically significant differences were observed in the yield strength values (see Supplementary Material 1.2 and Supplementary Figure S5). Nevertheless, the general mechanical behavior of the pillars remained the same regardless of the layer thickness or printing direction (Figures 3A,B). Moreover, the pillars exhibited no signs of buckling during the compression tests, as detailed by the DIC data in Figures 3A,B. The Young’s modulus and yield strength for the pillars composed of IP-Q and Grey resin were found to be comparable, as illustrated in Figure 3C.

### 3.2 Torsion testing

#### 3.2.1 Experimental torsion testing on solid grey resin torsion specimens

**FIGURE 4:**
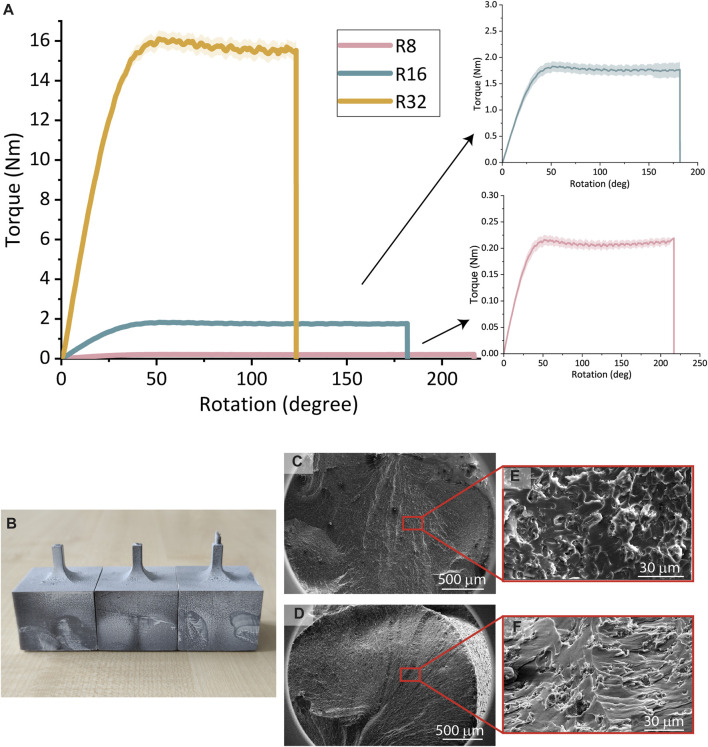
**(A)** The torque vs rotation curves of the torsion specimens 8R, 16R, and 32R printed from Grey resin. **(B)** The optical image of the fracture angles of the 8R torsion specimens. **(C, E)** SEM images of the 8R flat fracture surfaces. **(D, F)** SEM images of the 8R inclined fracture surface.

The largest torsion specimen (32R) failed at the lowest angle of rotation (123.2° ± 9.0°) and the highest applied torque (15.43 ± 0.39 Nm), while the smallest torsion specimens (8R) failed at the highest angle of rotation (216.9° ± 5.4°) and the lowest applied torque (0.21 ± 0.01 Nm) (Figure 4A). The 16R torsion specimens failed at a rotation of 181.7° ± 27.0° and a torque of 1.78 ± 0.08 Nm (Figure 4A). All the specimen types experienced failure in the cylindrical shaft close to the junction with the cuboid (Supplementary Materials 1.3 and Supplementary Figure S3). Among the 8R specimens, one exhibited a fracture angle of ≈44°, while the other two had a fracture angle of ≈0° (Figure 4B). The hackles on the inclined fracture surface appeared to radiate from a single point of origin, whereas the flat angle surface showed visible hackles without a single point of origin. The inclined-angle surface displayed elongated markings and relatively larger smooth sections within the roughness of the surface, in contrast to the flat-angle surface which exhibited more uniformly sized round markings (Figures 4C–F).

#### 3.2.2 Experimental and FEA simulation of the solid and porous grey resin torsion specimens

##### 3.2.2.1 Torque vs rotation

**FIGURE 5:**
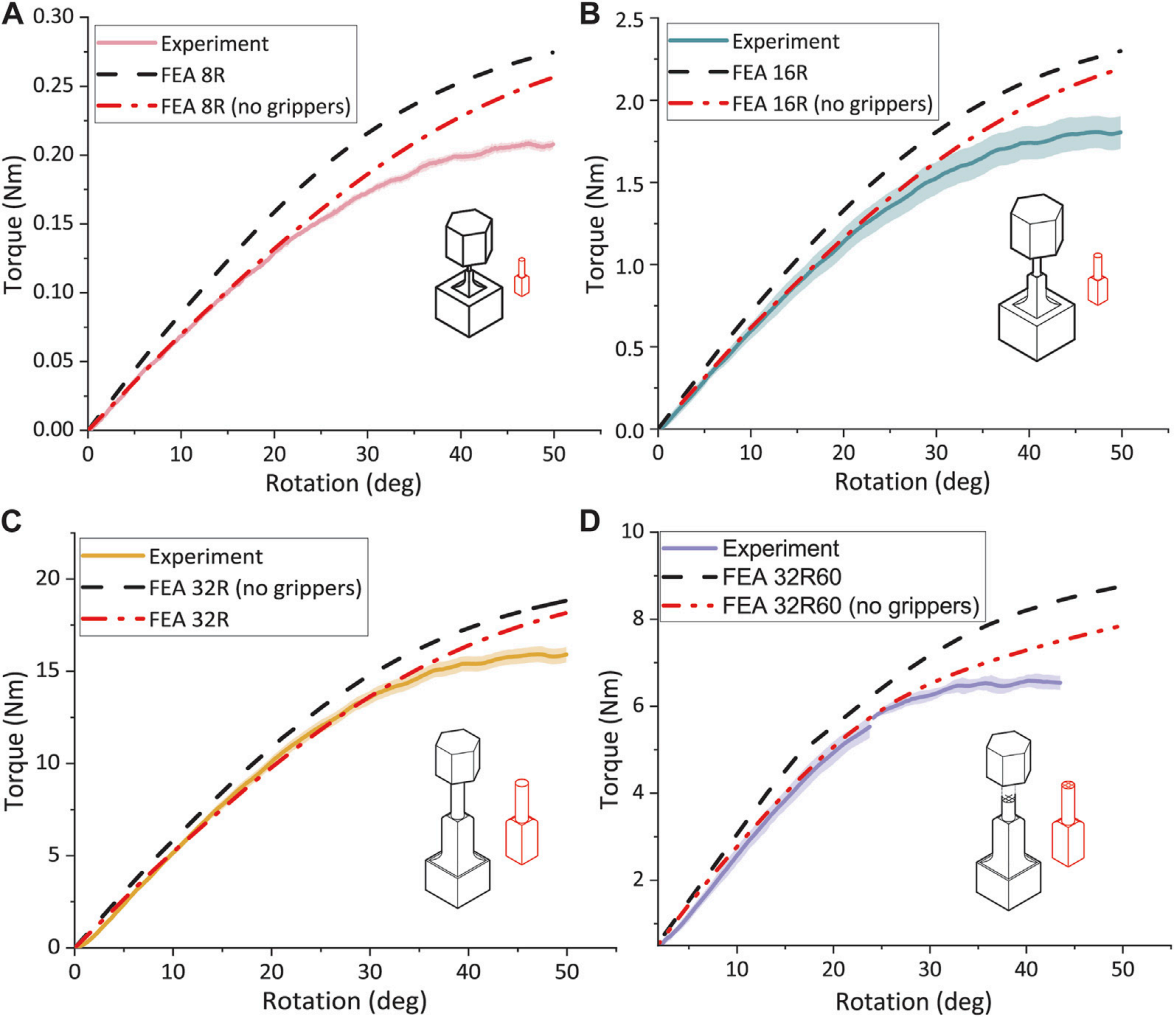
Torque vs rotation graph for all the torsion specimens. The experimental and FEA data for the **(A)** 8R, **(B)** 16R, **(C)** 32R, and **(D)** 32R60 torsion specimens with and without the grippers.

FEA simulation and experimental torsion testing on specimens 8R, 16R, and 32R confirmed that torque increased with the specimen size. The torque for the 16R specimens was approximately 8.5 times greater than that of the 8R torsion specimens and approximately 8.7 times lower than that of the 32R specimens. At 15° rotation, the measured torque values for the 8R, 16R, and 32R specimens were 85.6% ± 5.0%, 84.6% ± 5.1%, and 89.2% ± 2.8% of the torque value resulting from their corresponding FEA models, respectively. The difference between the measured and FEA-predicted values of torque increased with the rotation. For example, at 50° rotation, the torque measured for the 8R, 16R, and 32R torsion specimens were 77.1% ± 2.6%, 78.5% ± 3.5%, and 83.6% ± 2.2% of their corresponding FEA-predicted values, respectively. The FEA-predicted torque values of the torsion specimens were lower than the FEA-predicted torque values of the enlarged implants without grippers and were more similar to the experimental data. FEA simulations for all the sizes of the R design indicated that higher torques were needed to achieve the same rotation as in the torsion specimens (Figures 5A–C). However, as the size of the specimens increased, the difference between the FEA-predicted torque values of the torsion specimens and that of the R implants decreased, with the grippers playing a minor role in the final response of the specimens under torsion. A comparison between the torque-rotation values of the porous 32R60 specimens and the FEA simulations revealed that the specimens failed at the rotation angle of 38 ± 10°, which was approximately 3.2 times lower than the rotation angle corresponding to the failure point of the 32R specimens (Figures 5C,D). Within the linear part of the curve, the results of the FEA simulations and the experiments were in good agreement. For instance, at 15° rotation, the measured torque value for the porous 32R specimens was 98.9% ± 2.8% of the corresponding FEA values (Figure 5D).

##### 3.2.2.2 Shear stress vs shear strain

**FIGURE 6:**
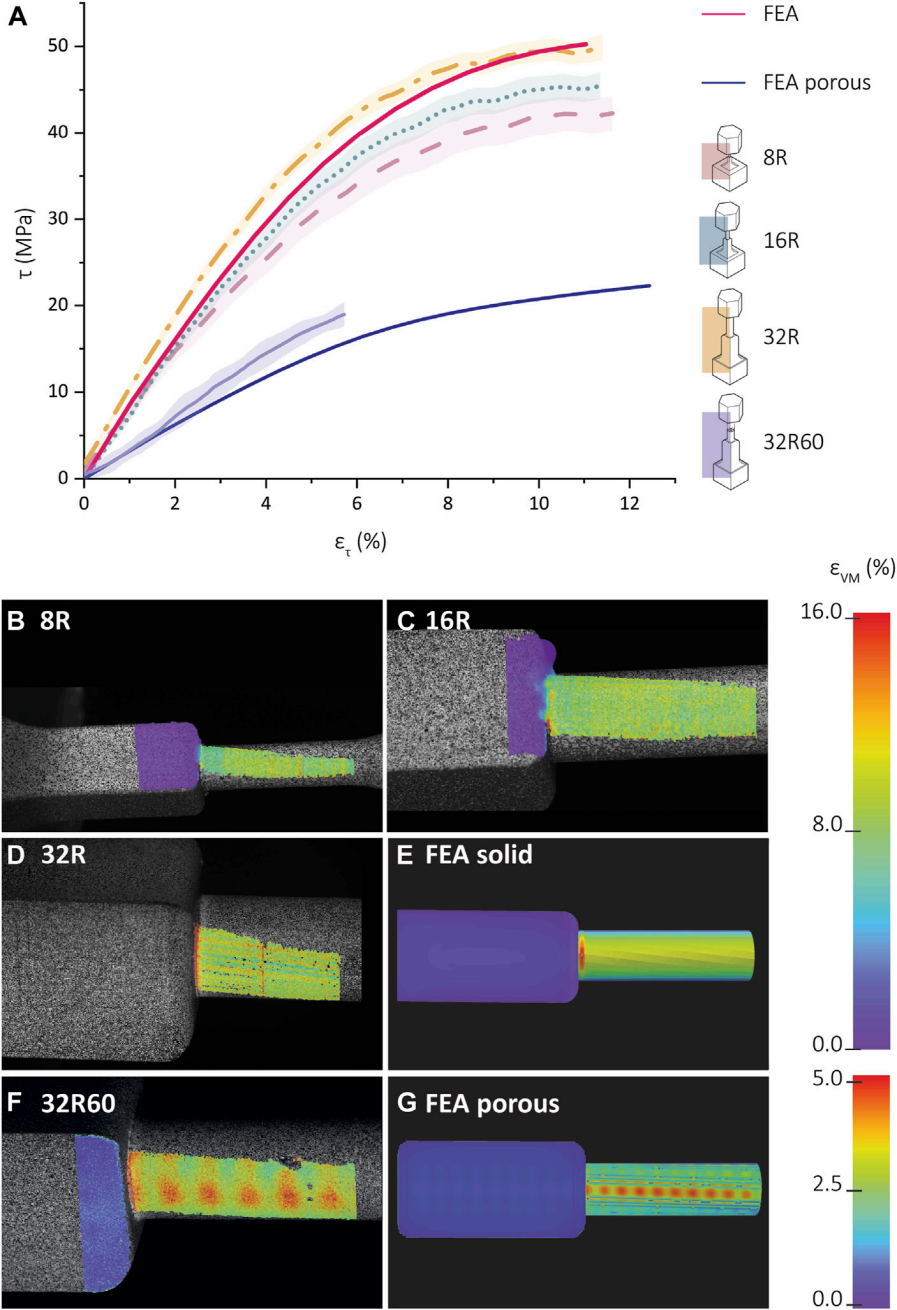
Shear stress-shear strain curves and full-field strain measurement using digital image correlation (DIC). **(A)** Shear stress vs shear strain for the SLA-printed torsion specimens printed from Grey-resin and the corresponding FEA data (red). The experimental data is averaged and is presented as mean ± standard deviation (shadowed lines). The DIC-measured distribution of the effective strain (von Mises) for the **(B)** 8R, **(C)** 16R, **(D)** 32R, and **(E)** FEA model of the scaled-up R torsion specimen. Color map strain: 0%–16%. **(F)** The FEA-predicted values of the effective strain (von Mises) for the porous 32R60 scaled-up torsion specimen. **(G)** The distribution of the FEA-predicted values of the effective strain (von Mises) for the porous torsion specimen. Color map strain: 0%–5%.

Comparing the shear stress-stain values between the FEA torsion specimens and experimental data for the 8R, 16R, and 32R specimens prior to the 0.2% yield (50° rotation) revealed that the shear stresses of the 8R and 16R specimens were lower than those predicted by the FEA models (Figure 6A). However, the shear stress values of the 32R specimens were higher than those obtained from the FEA simulations. Calculation of the shear modulus using the linear part of the shear stress-shear strain graph resulted in shear modulus values of 670.0 ± 34.0 MPa, 772.2. ± 30.6 MPa, and 867.1 ± 36.7 MPa, for the 8R, 16R, and 32R specimens, respectively. Based on Eq. (7), the theoretical value for the shear modulus is 896.1 MPa. The DIC strain distribution analysis for the 8R and 16R specimens revealed very low stress values near the junction (Figures 6B,C). However, the 32R specimens displayed the highest stress values at the junction between the cylindrical shaft and the cube (Figure 6D). The FEA strain distribution also showed similar values and behavior (Figure 6E). A comparison between the strain distributions measured by DIC for the porous 32R60 specimens and the corresponding FEA results revealed that the highest strain values were observed at the junction, as well as on the checkered patterns present on the cylinder, corresponding to the pores of the inner lattice structure (Figures 6F,G).

##### 3.2.2.3 FEA simulation of IP-Q implants

**FIGURE 7:**
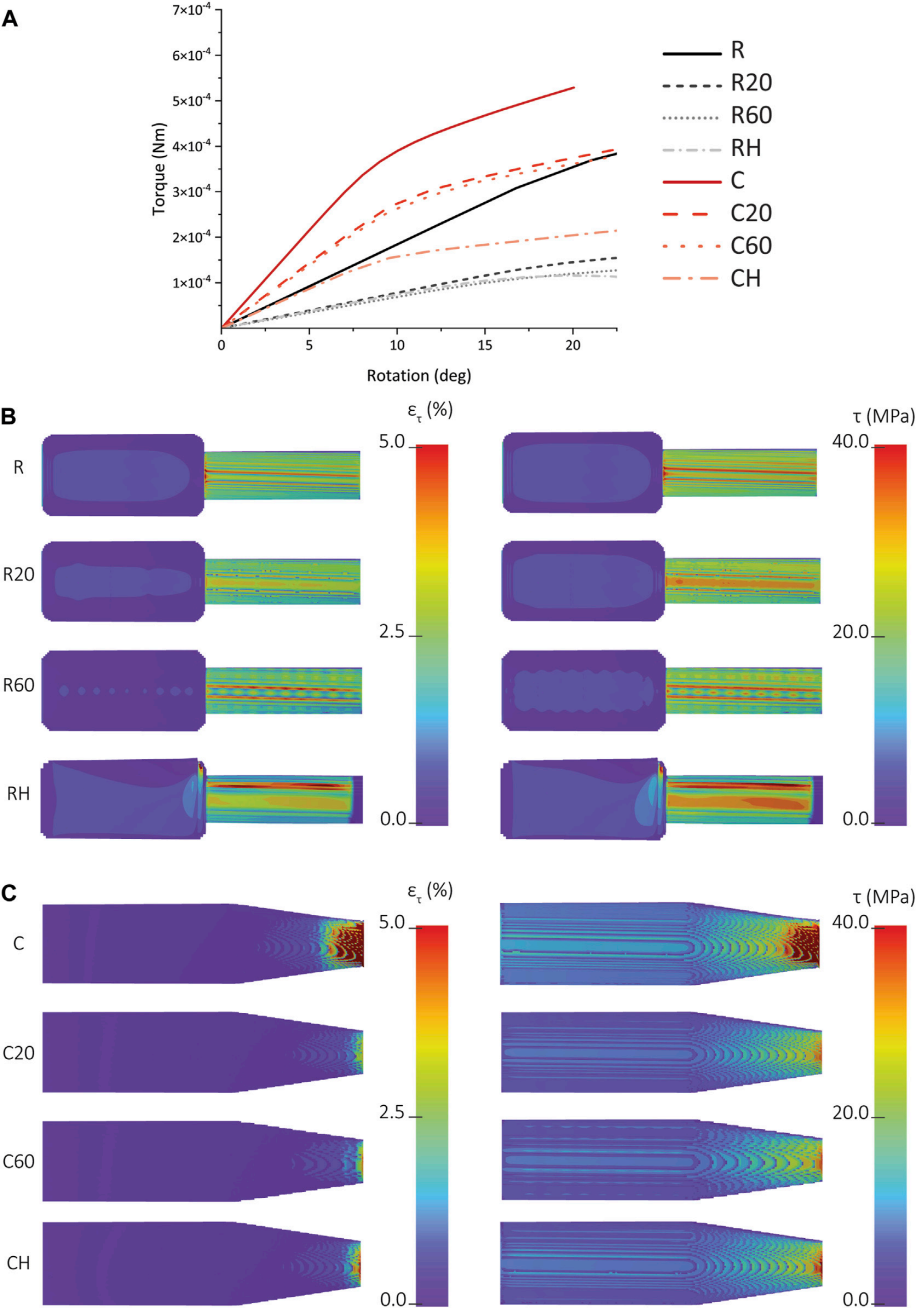
**(A)** The FEA-predicted torque vs rotation for the solid, porous, and hollow types of cochlear implants made from IP-Q (*i.e.*, R, R20, R60, RH, C, C20, C60, and CH). **(B)** The FEA-predicted values of the shear stress (on the left) and shear strain (on the right) corresponding to a rotation of 15° for the R, R20, R60, and RH specimens. **(C)** The FEA-predicted values of the shear stress (on the left) and shear strain (on the right) corresponding to a rotation of 15° for the C, C20, C60, and CH specimens.

A comparison between the FEA-predicted torque-rotation curves of the R, R20, R60, and RH IP-Q implants showed that much higher torque is required for the R specimens to achieve the same rotation angle, while there was a smaller difference between the torque values required for achieving the same rotation angle between the R20 and R60 implants. The shear stress and shear strain distribution measured for the R, R20, and R60 specimens at a rotation of 15° indicated the stress and strain values experienced by the cuboid part are close to zero, and the entire stress and strain distribution was limited to the cylindrical part (Figure 7B). In the case of the R specimens, the maximum shear stress and strain occurred at the junction. However, these observations were not valid for either of the porous designs, namely, R20 and R60. For R60, a discernible checkered pattern was observed within the cylindrical region, representing the presence of pores within the structure (Figure 7B). Additionally, two distinct lines were identified along the longitudinal axis of the cylinder, aligning with the regions of elevated shear strain and shear stress. However, the checkered patterns were not visible on the R20 design. Nonetheless, a broad line was discerned along the longitudinal axis of the cylinder, corresponding to the regions of maximum shear stress and strain within the structure. A comparison between the FEA-predicted torque-rotation curves of the C, C20, C60, and CH IP-Q implants showed that much higher torque is required for the C specimens to achieve the same rotation angle, while there was no significant difference between the torque values required for the C20 and C60 implants (Figure 7A). More specifically, the torque needed to rotate the C60 specimens by 15° was 97% of the torque needed to rotate the C20 specimens (Figure 7A). The distribution of the shear strain was similar for all the designs (Figure 7C). The C specimens had the highest maximum strain of the three designs, while C20 had the lowest maximum strain (Figure 7C). The shear strain was concentrated at the tip of the specimens, where the diameter of the design was the smallest (Figure 7C). A comparison between the results of the torsion tests on the two different designs revealed that, within each porous group, the C design exhibited greater stability and required higher torque to achieve the same amount of rotation as compared to the R design (Figure 7A). For instance, at a rotation angle of 15°, the torques required to rotate the C, C60, and C20 specimens were respectively 1.6, 3.1, and 2.7 times higher than the torques required to rotate the R, R20, and R60 specimens (Figure 7A).

### 3.3 Raman spectroscopy

The Raman spectra of the uncured and cured IP-Q exhibited characteristic peaks at 1,590 cm^-1^, 1,635 cm^-1^, and 1715 cm^-1^, associated with aromatic ring vibrations, double bond (C=C) stretching vibrations, and carbonyl (C=O) stretching vibrations, respectively. The peak intensity of the carbonyl (C=O) vibration did decrease upon curing. However, the peak intensity of the double bond (C=C) at 1,635 cm^-1^ and the aromatic ring vibration at 1,590 cm^-1^ decreased in the cured specimens. In the case of the uncured and cured Grey resin specimens, a prominent peak at 2,952 cm^-1^ corresponding to C-H stretching vibration was observed. Additionally, the uncured Grey resin exhibited peaks at 1715 cm^-1^ (carbonyl C=O stretching vibration) and 1,640 cm^-1^ (presence of monomers).

## 4 Discussion

The aim of the current study was to mechanically characterize the newly developed 3D printed cochlear implants designed for local drug delivery. This characterization considered the method of fabrication and implantation, factors which can influence their effectiveness as a permanent drug delivery system. Due to the unique size and complex shape of these implants, mechanical testing on such implants with standard testing equipment was challenging and had received limited attention. Consequently, the development of a novel approach was necessary, incorporating multi-scale *in silico* and *ex silico* methods to achieve a comprehensive mechanical characterization of cochlear implants. To address size and shape limitations, enlarged models of cochlear implants were simulated for torsion using FEA models, which were further validated through torsion tests. By extrapolating the validated *in silico* model to real-size implants, the study enabled the mechanical characterization of various cochlear implant designs, facilitating the selection of the most suitable one for cochlear implantation. Moreover, the development of this new method provided an opportunity to investigate some additional parameters related to the 3D printing techniques employed and the materials utilized.

### 4.1 Effect of layer thickness and print orientation on the Young’s modulus of Grey resin

Compression testing was performed on the pillars printed with Grey resin using different printer settings. Two different layer thicknesses (25 and 50 μm) and two different print orientations (horizontal and lateral layers) were used. The Young’s modulus was determined for each of these four conditions. In the case of the horizontally printed pillars, the Young’s modulus of the pillars printed with a 25 μm layer thickness was 2.51 ± 0.13 GPa, while for the 50 μm layer thickness, it was 2.50 ± 0.10 GPa. The laterally printed pillars had a Young’s modulus of 2.51 ± 0.050 GPa for the pillars printed with a 25 μm layer thickness and 2.33 ± 0.20 GPa for the 50 μm layer thickness pillars. The lack of difference in the Young’s modulus between the pillars with different layer thicknesses was unexpected, as previous research on specimens with different layer thicknesses has observed differences in the tensile strength and the Young’s modulus (Chockalingam et al., 2008; Wang et al., 2020). The literature is divided on whether different print orientations should affect the Young’s modulus of the material. Some studies have found that SLA printed parts are broadly isotropic materials, and print orientation does not significantly affect the Young’s modulus (Hague et al., 2004; Dizon et al., 2018; Aravind Shanmugasundaram et al., 2020; Cosmi and Dal Maso, 2020), which is in line with our findings. However, other studies have reported a dependence of the mechanical properties on the printing direction (Wang et al., 2020; Dulieu-Barton and Fulton, 2000; Saini et al., 2020). It is worth noting that most of these tests are performed using tensile testing, making it difficult to directly compare them with the compression tests we conducted here. Therefore, a more comprehensive study investigating the anisotropy of SLA materials, possibly employing different mechanical tests, is necessary to draw definitive conclusions.

### 4.2 Failure mode analysis of Grey resin

One of the crucial aspects of a material’s mechanical behavior is its failure mode, which can generally be categorized as either ductile or brittle (Hayes et al., 2015). In this study, experimental torsion testing was conducted on Grey resin torsion specimens until failure to determine the material’s failure mode. Several factors were considered, including the shape of torque *versus* rotation curves, the failure angles of the torsion specimens, and the SEM images of the fracture surface. Analysis of the experimental torque *versus* rotation curves for all three scales of the specimens revealed that the material yielded after approximately 50° of rotation, with the torque reaching a plateau as rotation increased. This behavior indicates a ductile material, as it exhibits a large area under the stress-strain curve due to continued deformation after the yield point (Hayes et al., 2015), and implies that the torsion specimens experienced a ductile failure mode. Next, the failure angles of the specimens were examined. In ductile materials, torsion failure typically occurs along the plane of maximum shear stress since they are weaker in shear, which is perpendicular to the structure’s axis. In contrast, brittle materials are weaker in tension, and torsion failure tends to occur along the plane of maximum tension, resulting in a 45° angle (Hailu et al., 2021; Sadaghian et al., 2022). Out of the ten analyzed failure angles, three (two 8R and one 16R torsion specimens) exhibited a failure angle perpendicular to the axis of the structure, indicating shear failure. The remaining six measured specimens had failure angles ranging between 31° and 44°, suggesting that these specimens were weaker in tension than in shear (Supplementary Table S1). The variation in failure rotational angles among the specimens is associated with the differences in specimen sizes. Furthermore, SEM images were captured of the fracture surfaces of two 8R torsion specimens: one with a flat failure angle and one with an inclined failure angle. Both specimens displayed a rough surface with visible micropatterning and fibrillations, although the specimen with the flat angle exhibited overall smaller micropatterning compared to the inclined-angle specimen. A study examining the fracture surfaces of tensile test specimens made using SLA from an acrylate polymer MD-R001CR (ApplyLabWork, Torrance, United States) concluded that specimens with a highly ductile stress-strain curve exhibited a smooth fracture surface, while specimens with a more brittle stress-strain curve had rougher fractures (Quagliato et al., 2022). This observation does not hold for our torsion specimens made out of Grey resin, as they demonstrate a ductile stress-strain curve but exhibit a rough fracture surface. An exact comparison between the results of the previous study and our study can, therefore, not be made since the type of the material seems to influence the mechanical and fractural behavior more than the manufacturing technique. In summary, the various aspects of the failure mode analysis cannot predominantly conclude a brittle or ductile failure in the Grey resin material. Conducting additional torsion testing to examine the post-yield plastic behavior of the material would help clarify this inconsistency. Understanding the failure mode of Grey resin is essential for informing its potential applications.

### 4.3 Comparative analysis of experimental and FEA torsion testing results

For both solid and porous specimens, the experimental torsion tests conducted on the torsion specimens made from Grey resin were compared with the torsion tests simulated using FEA. When comparing the FEA-predicted and experimental torque-rotation curves for the solid specimens, good agreement was observed for the elastic-linear part of the curve until yielding. The porous 32R60 specimen also showed good agreement between the experimental and FEA curves, although the porous specimens failed earlier than their solid counterparts, which was not captured by the FEA models. However, significant differences were observed in the post-yield behavior between the FEA simulations and experimental results for the solid R specimens. The stress-strain curve obtained from compression testing showed strain hardening, which was not observed during torsion testing. Therefore, using compression stress-strain curves as input data for FEA may not be accurate for simulating the post-yield behavior observed in the torsion tests. Comparing the solid upscaled R and solid torsion specimens through FEA, we found that the R exhibits higher torques for all the three sizes, indicating that the inclusion of the additional grippers lowers the torque-rotation curve which is translated into a decrease in the total needed energy to failure. The difference between the experimental and simulation data increased with the degree of rotation, with a smaller difference observed for the 32R torsion specimens as compared to the 8R and 16R torsion specimens. This apparent dependence on the size might be due to the relatively greater added length of the structures in the 8R torsion specimens as compared to the 32R torsion specimens. The shear stress-strain curves were approximated by considering only the cylindrical part of the specimen, as it experiences most of the generated stress and strain due to its smaller diameter as compared to the cubic part. Good agreement between the experimental and FEA-predicted curves was observed in the linear elastic region. Similar to the torque-rotation curves, a trend was observed where the 8R and 16R torsion specimens deviated more from the FEA shear stress-strain curve compared to 32R torsion specimens. The experimentally measured and FEA-predicted strain distribution in both solid and porous torsion specimens presented similar patterns. For the porous 32R torsion specimens, both showed a checkered pattern with higher strain areas corresponding to the inner unit of the lattice structure. The experimental DIC distribution exhibits a similar pattern as the FEA strain distribution, with higher strain areas occurring between the struts of the inner lattice structure. The strain distribution of the Grey resin specimens revealed that the higher strain areas observed in the porous specimens corresponded to the inner unit of the lattice structure, which had a pore size of approximately 1.9 × 1.9 × 1.9 mm³. In the FEA simulations, the higher strain areas within the cylindrical part were located between the struts of the inner lattice structure. Comparing the experimental data obtained for the Grey resin with the FEA models, good agreement was found in the linear-elastic region for both torque-rotation and stress-strain curves, as well as strain distributions. This indicates that Grey resin can be accurately modeled with FEA using compression data as input in the linear-elastic region.

### 4.4 Analysis of FEA modeling: Grey resin vs IP-Q

SLA and 2PP are additive manufacturing techniques based on the principle of polymerization. While SLA involves the absorption of a single photon, 2PP requires the absorption of two photons (Pagac et al., 2021; Waheed et al., 2016). Due to the similarities in the manufacturing process and mechanical properties of Grey resin (printed with SLA) and IP-Q (printed with 2PP), it is expected that the mechanical behavior of the printed structures would exhibit similar characteristics. The measured Young’s moduli of Grey resin and IP-Q were similar (2.50 GPa and 2.78 GPa, respectively). Validation of the FEA model using torsion testing on IP-Q structures was not possible due to the limited size that can be achieved with 2PP printing. However, with Grey resin, sufficiently large specimens were printed using SLA to conduct torsion testing, allowing for experimental validation of the FEA model. The linear regions of both experimental and FEA-predicted data demonstrated good agreement (Figure 6), enabling the extrapolation of the FEA simulations to predict the linear behavior of the ear implants made from IP-Q. In the FEA simulations, the input data for IP-Q, including the Young’s modulus and stress-strain curve, were obtained from compression testing of a solid cylindrical structure made from IP-Q, similar to the process for Grey resin. However, the IP-Q input data was derived from a single compression experiment, unlike Grey resin data which was derived from three compression experiments on distinct pillars. Consequently, the reliability of the IP-Q input data is comparatively lower, but the measured values are in line with the values reported in the literature (Schweiger et al., 2022). Despite their distinct Young’s moduli and yield points, the shear stress-shear strain curves predicted by the FEA models for both materials presented similar overall trends. This similarity suggests that the torsional behavior of IP-Q can be accurately simulated using FEA techniques similar to those applied for Grey resin. Utilizing FEA modeling to characterize IP-Q could reduce the reliance on specialized equipment and facilitate the mechanical testing of more complex designs.

### 4.5 FEA of mechanical behavior: R vs C implant designs

In the case of IP-Q, both the R and C cochlear implant designs were simulated using FEA models, considering both porous and non-porous variations. Introducing porosity to the designs resulted in decreased stiffness and mechanical strength for each implant. Although the difference in the stiffness and mechanical strength between the identical designs with different pore sizes (*i.e.*, 20 and 60 µm) was not substantial, the difference in stiffness and mechanical strength between the different types of implants (*i.e.*, R and C) was notable. At a rotation angle of 15°, the C design exhibited a mere 3% reduction in stiffness and mechanical strength, whereas the R design experienced a more pronounced decrease of 13% (Figure 7) when compared to their solid versions, respectively. This implies that the porous structure had a greater impact on the mechanical properties of the R as compared to the C. The shear stress-strain distribution of the porous R displayed a distinct pattern for both the 20 and 60-pore size variants, which was absent in the solid R. This pattern suggests the presence of stress concentrations arising from the inner lattice structure of the square unit, particularly near the connections between the struts where sharp transitions occur (Al-Ketan et al., 2018). Conversely, the porous C did not exhibit such patterns. The shear stress-shear strain distributions of both solid and porous C implants presented similarities. Notably, the C design exhibited higher stiffness and mechanical strength as compared to R. This verifies our attempt to enhance the mechanical performance of the R-type implant. To achieve this, we introduced a tapering angle at the interface between the drug reservoir and the tip of the implant, which led to the creation of the C-type implant. From a mechanical viewpoint, the design of the C-type implant is more advantageous while preserving the overall dimensions (Gehrke et al., 2016).

### 4.6 Polymerization of IP-Q and Grey resin during 2PP and SLA printing

Existing studies have established a correlation between the polymerization that occurs during 3D printing and its influence on the mechanical properties of the resulting structures (Schweiger et al., 2022; LaFratta and Baldacchini, 2017). Raman spectroscopy allows for the analysis of distinct peaks associated with different intramolecular bonds, providing insights into the molecular structure changes during polymerization. Therefore, the polymerization process during 2PP printing and SLA printing was investigated by conducting Raman spectroscopy on unpolymerized and polymerized IP-Q and Grey resin. The C=C bond, typically present at 1,640 cm^-1^, showed lower intensity in the polymerized IP-Q and Grey resin specimens as compared to the unpolymerized specimens. This decrease in the C=C bond peak is expected for photocurable resins undergoing polymerization (Jiang et al., 2014; Pianelli et al., 1999). Furthermore, the unpolymerized IP-Q resin exhibited a peak at 1,590 cm^-1^, which is associated with the aromatic ring present in IP-Q. This peak is specific to IP-Q and is not observed (at the same intensity) in most other photoresins (Saldívar et al., 2022; Pianelli et al., 1999; Jiang et al., 2014; Liu et al., 2018; Bauer et al., 2019). The decrease in the intensity of the aromatic ring peak after polymerization suggests its potential involvement in the polymerization process of IP-Q, a resin specifically designed for high-speed fabrication. Since the aromatic ring also contains C=C bonds, its contribution may influence the intensity of the C=C bond peak, consequently impacting the polymerization. Albeit, no parametric study of the 2PP printing parameters that influence polymerization, hence the mechanical properties of IP-Q was performed, IP-Q was chemically characterized for the printing conditions suitable for the fabrication of the cochlear implants. Further investigations on IP-Q resin can provide additional insights into the specific mechanisms of polymerization during the printing of this innovative resin. For the Grey resin, there is a peak at 1715 cm^-1^ that decreases significantly after printing, associating it with the polymerization process. The findings indicated a high DC and a high degree of printing accuracy and are in line with a previous study on commercially available methacrylate-based resins used in SLA printing (Băilă and Tonoiu, 2022).

## 5 Conclusion

In conclusion, we developed a methodology to characterize the mechanical behavior of cochlear implants of relevant sizes for humans. We used FEA models to validate the results of the experiments. The use of 2PP with the novel resin IP-Q for printing the ear implants, along with the scaled-up SLA-printed cochlear implants made from Grey resin enabled us to study their mechanical properties. The FEA simulations accurately captured the linear behavior of the ear implants until the yield point, while only utilizing compression data. This validation facilitated the use of these FEA models to study the torsion testing of the real-size implants made from IP-Q using only compression data. Comparing both ear implant designs, the cylindrical implants exhibited higher stiffness and mechanical strength than the rectangular ones. Although the size of the pores (*i.e.*, 20 and 60 µm) did not have a significant effect on the mechanical performance of the implant, they could have a major impact on other factors, such as drug storage and diffusion in the final design. From a mechanical perspective, the cylindrical design emerges as the preferred option. Characterizing the mechanical properties of these distinct inner ear implant designs using the IP-Q resin represents a novel contribution. Additionally, this study marks the first time such ear implants have been mechanically characterized using this approach. The obtained data will contribute to the assessment of the implants’ ability to withstand mechanical stress during implantation, helping to determine whether such types of ear implants are suitable for the potential treatment of inner ear disorders. Future research directions may involve finding alternative methods to model post-yield behavior and exploring various geometries of the inner structures. Overall, this study provides valuable insights into the mechanical behavior of cochlear implants and lays the groundwork for further advancements in this field.

## Data Availability

The raw data supporting the conclusions of this article will be made available by the authors, without undue reservation.

## References

[B1] Al-Ketan O. Rezgui R. Rowshan R. Du H. Fang N. X. Abu Al-Rub R. K. (2018). Microarchitected stretching-dominated mechanical metamaterials with minimal surface topologies. Adv. Eng. Mater 20 (9), 1800029. 10.1002/adem.201800029 10.1002/adem.201800029 | Google Scholar

[B2] Arasaratnam P. Sivakumaran K. S. Tait M. J. (2011). True stress-true strain models for structural steel elements. ISRN Civ. Eng. 2011, 1–11. 10.5402/2011/656401 10.5402/2011/656401 | Google Scholar

[B3] Aravind Shanmugasundaram S. Razmi J. Mian M. J. Ladani L. (2020). Mechanical anisotropy and surface roughness in additively manufactured parts fabricated by stereolithography (SLA) using statistical analysis. Materials 13 (11), 2496. 10.3390/ma13112496 PubMed Abstract | 10.3390/ma13112496 | Google Scholar 32486137 PMC7321476

[B4] Ast J. Mohanty G. Guo Y. Michler J. Maeder X. (2017). *In situ* micromechanical testing of tungsten micro-cantilevers using HR-EBSD for the assessment of deformation evolution. Mater Des. 117, 265–266. 10.1016/j.matdes.2016.12.052 10.1016/j.matdes.2016.12.052 | Google Scholar

[B5] ASTM International (2019). D695 − 15 standard test method for compressive properties of rigid plastics 1. 10.1520/D0695-15 10.1520/D0695-15 | Google Scholar

[B6] ASTM International (2020). E143 − 20 standard test method for shear modulus at room temperature. 10.1520/E0143-20 10.1520/E0143-20 | Google Scholar

[B7] Băilă D. I. Tonoiu S. (2022). Properties of photo-curable polyurethane resins used in SLA manufacturing. IOP Conf. Ser. Mater Sci. Eng. 1268 (1), 012006. 10.1088/1757-899X/1268/1/012006 10.1088/1757-899X/1268/1/012006 | Google Scholar

[B8] Bauer J. Guell Izard A. Zhang Y. Baldacchini T. Valdevit L. (2019). Programmable mechanical properties of two‐photon polymerized materials: from nanowires to bulk. Adv. Mater Technol. 4 (9), 1900146. 10.1002/admt.201900146 10.1002/admt.201900146 | Google Scholar

[B9] Chockalingam K. Jawahar N. Chandrasekar U. Ramanathan K. N. (2008). Establishment of process model for part strength in stereolithography. J. Mater Process Technol. 208 (1–3), 348–365. 10.1016/j.jmatprotec.2007.12.144 10.1016/j.jmatprotec.2007.12.144 | Google Scholar

[B10] Cosmi F. Dal Maso A. (2020). A mechanical characterization of SLA 3D-printed specimens for low-budget applications. Mater Today Proc. 32, 194–201. 10.1016/j.matpr.2020.04.602 10.1016/j.matpr.2020.04.602 | Google Scholar

[B11] Dizon J. R. C. Espera A. H. Chen Q. Advincula R. C. (2018). Mechanical characterization of 3D-printed polymers. Addit. Manuf. 20, 44–67. 10.1016/j.addma.2017.12.002 10.1016/j.addma.2017.12.002 | Google Scholar

[B12] Dulieu-Barton J. M. Fulton M. C. (2000). Mechanical properties of a typical stereolithography resin. Strain 36 (2), 81–87. 10.1111/j.1475-1305.2000.tb01177.x 10.1111/j.1475-1305.2000.tb01177.x | Google Scholar

[B13] Fu Z. Jiang L. Wardini J. L. MacDonald B. E. Wen H. Xiong W. (2018). A high-entropy alloy with hierarchical nanoprecipitates and ultrahigh strength. Sci. Adv. 4 (10), eaat8712. 10.1126/sciadv.aat8712 PubMed Abstract | 10.1126/sciadv.aat8712 | Google Scholar 30333993 PMC6184785

[B14] Gehrke M. Sircoglou J. Gnansia D. Tourrel G. Willart J. F. Danede F. (2016). Ear Cubes for local controlled drug delivery to the inner ear. Int. J. Pharm. 509 (1–2), 85–94. 10.1016/j.ijpharm.2016.04.003 PubMed Abstract | 10.1016/j.ijpharm.2016.04.003 | Google Scholar 27050866

[B15] Hague R. Mansour S. Saleh N. Harris R. (2004). Materials analysis of stereolithography resins for use in Rapid Manufacturing. J. Mater Sci. 39 (7), 2457–2464. 10.1023/B:JMSC.0000020010.73768.4a 10.1023/B:JMSC.0000020010.73768.4a | Google Scholar

[B16] Hailu Y. M. Nazir A. Lin S.-C. Jeng J.-Y. (2021). The effect of functional gradient material distribution and patterning on torsional properties of lattice structures manufactured using MultiJet fusion Technology. Materials 14 (21), 6521. 10.3390/ma14216521 PubMed Abstract | 10.3390/ma14216521 | Google Scholar 34772057 PMC8585127

[B17] Hao J. Li S. K. (2019). Inner ear drug delivery: recent advances, challenges, and perspective. Eur. J. Pharm. Sci. 126, 82–92. 10.1016/j.ejps.2018.05.020 PubMed Abstract | 10.1016/j.ejps.2018.05.020 | Google Scholar 29792920

[B18] Hayes M. D. Edwards D. B. Shah A. R. (2015). “Fractography basics,” in Fractography in failure analysis of polymers (Elsevier), 48–92. 10.1016/B978-0-323-24272-1.00004-0 10.1016/B978-0-323-24272-1.00004-0 | Google Scholar

[B19] Isaakidou A. Apachitei I. Fratila-Apachitei L. E. Zadpoor A. A. (2023). High-precision 3D printing of microporous cochlear implants for personalized local drug delivery. J. Funct. Biomater. 14 (10), 494. 10.3390/jfb14100494 PubMed Abstract | 10.3390/jfb14100494 | Google Scholar 37888159 PMC10607433

[B20] Jiang L. J. Zhou Y. S. Xiong W. Gao Y. Huang X. Jiang L. (2014). Two-photon polymerization: investigation of chemical and mechanical properties of resins using Raman microspectroscopy. Opt. Lett. 39, 3034–3037. 10.1364/OL.39.003034 PubMed Abstract | 10.1364/OL.39.003034 | Google Scholar 24978266

[B21] Jun T.-S. Sernicola G. Dunne F. P. E. Britton T. B. (2016). Local deformation mechanisms of two-phase Ti alloy. Mater. Sci. Eng. A 649, 39–47. 10.1016/j.msea.2015.09.016 10.1016/j.msea.2015.09.016 | Google Scholar

[B22] LaFratta C. Baldacchini T. (2017). Two-photon polymerization metrology: characterization methods of mechanisms and microstructures. Micromachines (Basel) 8 (4), 101. 10.3390/mi8040101 10.3390/mi8040101 | Google Scholar

[B23] Lin Z. Novelino L. S. Wei H. Alderete N. A. Paulino G. H. Espinosa H. D. (2020). Folding at the microscale: enabling multifunctional 3D origami‐architected metamaterials. Small 16 (35), 2002229. 10.1002/smll.202002229 10.1002/smll.202002229 | Google Scholar 32715617

[B24] Liu Y. Campbell J. Stein O. Jiang L. Hund J. Lu Y. (2018). Deformation behavior of foam laser targets fabricated by two-photon polymerization. Nanomaterials 8 (7), 498. 10.3390/nano8070498 PubMed Abstract | 10.3390/nano8070498 | Google Scholar 29986426 PMC6070906

[B25] Pagac M. Hajnys J. Ma Q. P. Jancar L. Jansa J. Stefek P. (2021). A review of vat photopolymerization Technology: materials, applications, challenges, and future trends of 3D printing. Polym. (Basel) 13 (4), 598. 10.3390/polym13040598 10.3390/polym13040598 | Google Scholar PMC7922356 33671195

[B26] Pianelli C. Devaux J. Bebelman S. Leloup G. (1999). The micro-Raman spectroscopy, a useful tool to determine the degree of conversion of light-activated composite resins. J. Biomed. Mater Res. 48 (5), 675–681. 10.1002/(SICI)1097-4636(1999)48:5<675::AID-JBM11>3.0.CO;2-P PubMed Abstract | 10.1002/(SICI)1097-4636(1999)48:5<675::AID-JBM11>3.0.CO;2-P | Google Scholar 10490681

[B27] Quagliato L. Yeon Kim S. Ryu S. C. (2022). Quasi-ductile to brittle transitional behavior and material properties gradient for additively manufactured SLA acrylate. Mater Lett. 329, 133121. 10.1016/j.matlet.2022.133121 10.1016/j.matlet.2022.133121 | Google Scholar

[B28] Sadaghian H. Khalilzadehtabrizi S. Farzam M. Dehghan S. (2022). Behavior of 3D-printed polymers under monotonic torsion – a database of 15 different materials. Addit. Manuf. 60, 103251. 10.1016/j.addma.2022.103251 10.1016/j.addma.2022.103251 | Google Scholar

[B29] Saini J. Dowling L. Kennedy J. Trimble D. (2020). Investigations of the mechanical properties on different print orientations in SLA 3D printed resin. Proc. Inst. Mech. Eng. C J. Mech. Eng. Sci. 234 (11), 2279–2293. 10.1177/0954406220904106 10.1177/0954406220904106 | Google Scholar

[B30] Saldívar M. C. Doubrovski E. L. Mirzaali M. J. Zadpoor A. A. (2022). Nonlinear coarse-graining models for 3D printed multi-material biomimetic composites. Addit. Manuf. 58, 103062. 10.1016/j.addma.2022.103062 10.1016/j.addma.2022.103062 | Google Scholar

[B31] Schweiger S. Schulze T. Schlipf S. Reinig P. Schenk H. (2022). Characterization of two-photon-polymerization lithography structures via Raman spectroscopy and nanoindentation. J. Opt. Microsystems 2 (3), 33501. 10.1117/1.jom.2.3.033501 10.1117/1.jom.2.3.033501 | Google Scholar

[B32] Szeto B. Chiang H. Valentini C. Yu M. Kysar J. W. Lalwani A. K. (2020). Inner ear delivery: challenges and opportunities. Laryngoscope Investig. Otolaryngol. 5 (1), 122–131. 10.1002/lio2.336 PubMed Abstract | 10.1002/lio2.336 | Google Scholar PMC7042639 32128438

[B33] Waheed S. Cabot J. M. Macdonald N. P. Lewis T. Guijt R. M. Paull B. (2016). 3D printed microfluidic devices: enablers and barriers. Lab. Chip 16 (11), 1993–2013. 10.1039/C6LC00284F PubMed Abstract | 10.1039/C6LC00284F | Google Scholar 27146365

[B34] Wang S. Ma Y. Deng Z. Zhang K. Dai S. (2020). Implementation of an elastoplastic constitutive model for 3D-printed materials fabricated by stereolithography. Addit. Manuf. 33, 101104. 10.1016/j.addma.2020.101104 10.1016/j.addma.2020.101104 | Google Scholar

[B35] Zhang Z. Li X. Zhang W. Kohane D. S. (2021). Drug delivery across barriers to the middle and inner ear. Adv. Funct. Mater 31 (44), 2008701. 10.1002/adfm.202008701 PubMed Abstract | 10.1002/adfm.202008701 | Google Scholar 34795553 PMC8594847

[B36] Zhao X. Strickland D. J. Derlet P. M. He M. r. Cheng Y. J. Pu J. (2015). *In situ* measurements of a homogeneous to heterogeneous transition in the plastic response of ion-irradiated ⟨1 1 1⟩ Ni microspecimens. Acta Mater 88, 121–135. 10.1016/j.actamat.2015.01.007 10.1016/j.actamat.2015.01.007 | Google Scholar

