# Supplementary material for: Multi-scale in silico and ex silico mechanics of 3D printed cochlear implants for local drug delivery
Source: Front Bioeng Biotechnol. 2024 Jan 31;11:1289299. doi: 10.3389/fbioe.2023.1289299 (PMC10865239; doi:10.3389/fbioe.2023.1289299)
Supplement: Supplementary file 1 [file DataSheet1.docx]

Supplementary Material

Multi-scale *ex silico* and *in silico* mechanics of 3D printed cochlear implants for local drug delivery

A. Isaakidou^1^*, M. Ganjian^1^, R. van Hoften^1^, M.C. Saldivar^1^, M.A. Leeflang^1^, A. Groetsch^2,3^, M. Wątroba^2^, J. Schwiedrzik^2^, M.J. Mirzaali^1^, I. Apachitei^1^, L.E. Fratila-Apachitei^1^*, A.A. Zadpoor^1^

^1^Department of Biomechanical Engineering, Faculty of Mechanical Engineering, Delft University of Technology (TU Delft), Mekelweg 2, 2628 CD, Delft, The Netherlands

^2^Empa, Swiss Federal Laboratories for Materials Science and Technology, Laboratory of Mechanics of Materials and Nanostructures, CH-3602 Thun, Feuerwerkerstrasse 39, Switzerland

^3^Department of Materials Science and Engineering, Henry Samueli School of Engineering, University of California, Irvine, CA 92697, US

*Correspondence:
A. Isaakidou, [A.Isaakidou@tudelft.nl](mailto:A.Isaakidou@tudelft.nl); L.E. Fratila-Apachitei, [E.L.Fratila-Apachitei@tudelft.nl](mailto:E.L.Fratila-Apachitei@tudelft.nl);

# Supplementary studies

## Convergence study of the FEA torsion model

Torsion testing was initially modeled on a simple cylinder with a diameter of 10 mm and a length of 20 mm. The output of the FEA simulation was the torque (reaction moment) and the shear stress of the cylinder after rotation. The assigned material properties (*E* = 2504 MPa, *ν* = 0.45) were obtained from the previously conducted compression tests for the Grey resin pillars. Two reference points (RP) were created, at the center of the top and bottom parts of the cylinder, namely RP-1 and RP-2. The top and bottom surfaces of the cylinder were coupled to these reference points to constrain the coupled nodes' motion to the reference point's rigid body motion. RP-2 was then encastered (*i.e.*, constraining all translations U or rotations UR) while RP-1 was set to rotate by 0.2 radians. The torsion was modeled in the linear region and the angle of twist (θ) was calculated as (13):

$\theta= \frac{TL}{GJ}$ (Eq. S1)

where *T* is the torque, *L* is the length of the cylinder, *G* is the shear modulus, and *J* is the second moment of area.

The shear modulus was calculated from the given material properties as (Eq. S2):

$E=2G(1+\nu)$ (Eq. S2)

To determine the size of the mesh required for accurate results, a mesh convergence study on three different types of mesh elements, namely on the eight-node brick element (C3D8), the ten-node tetrahedral element (C3D10), and the twenty-node brick element with reduced integration (C3D20R), was performed. The convergence study was performed for both the torque and shear stress. The theoretical values of the torque *T* = 8.48 Nm and shear stress *τ* = 43.17 MPa were used as boundary conditions for the simulations. The comparison was made by calculating the respective torque using the resulting rotation angle and Eq.1-6. We assumed convergence when the relative error with the theoretical values was below 5%. After processing the simulations, shear stress convergence was achieved with only 70 C3D8 elements, while 51414 C3D8 elements were required to converge for torque (Supplementary Figure 1A-B). Similarly, 17755 C3D10 and 4500 C3D20R elements were required for shear stress, and 3477 C3D10 and 2420 C3D20R elements for torque, respectively. From these results, the C3D10 element models required the least CPU time (*i.e.*, 663 seconds per simulation), making them the most efficient simulations (Supplementary Figure 1C). Therefore, the C3D10 mesh was used to model the torsion tests. The refinement of the number of elements was based on the shear stress convergence with a threshold of 1.0% and resulted in 25,000 elements. Therefore, the C3D10 mesh was used to model the torsion tests. The refinement of the number of elements was based on the shear stress convergence with a threshold of 1.0% and resulted in 25,000 elements.

## Statistical Exploration and Analysis

Shapiro-Wilk normality tests were performed on the datasets for the vertical and horizontal printed pillars in the two different layer thicknesses (25 and 50 μm) as part of the statistical exploration of the data before the statistical analysis. The normality test results are presented in QQ-normality plots (Supplementary Figure 3) and the resulting p-values are presented in Supplementary Table 2. The one-way ANOVA statistical test results are presented in Supplementary Figure 4.

## Failure modes of solid Grey resin torsion specimens

The torsion specimens exhibited different fracture angles. Those fracture angles have been either flat or inclined (Supplementary Table 1). All torsion specimens failed very close to the junction between the cubic and the cylindrical shaft or in the cylindrical shaft (Supplementary Figure 2).

# Supplementary Figures and Tables

## Supplementary Figures


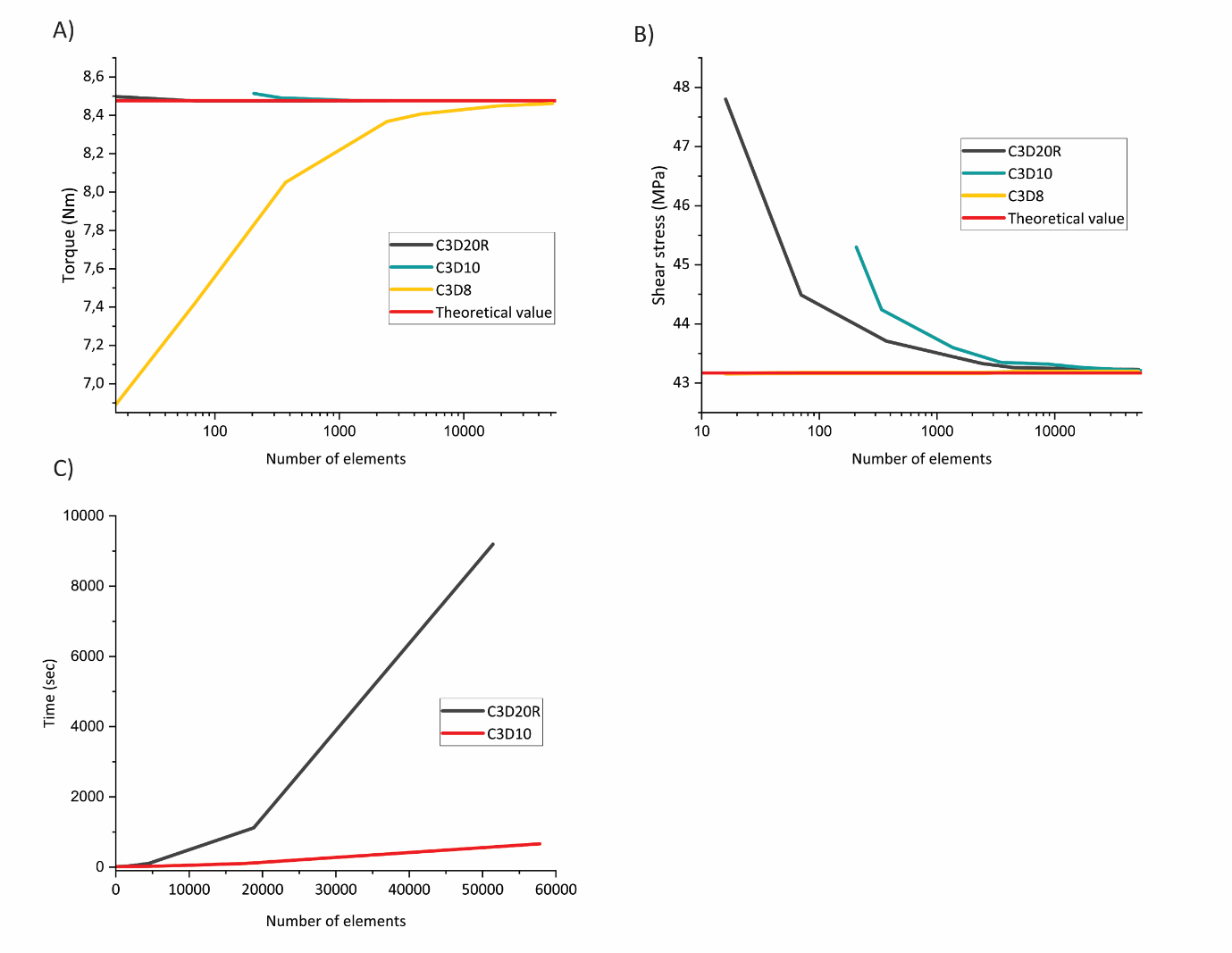


**Supplementary Figure 1.** A convergence study of the torque and shear stress calculated by the FEA models for a cylinder: A) The torque vs. the number of elements in the mesh for tetrahedral (C3D10) and hexahedral (C3D8, C3D20R) elements. B) The shear stress vs. the number of elements in the mesh for tetrahedral (C3D10) and hexahedral (C3D8, C3D20R) elements. C)The computational time (s) required to compute the model using the C3D10 or C3D20R type of elements.

**
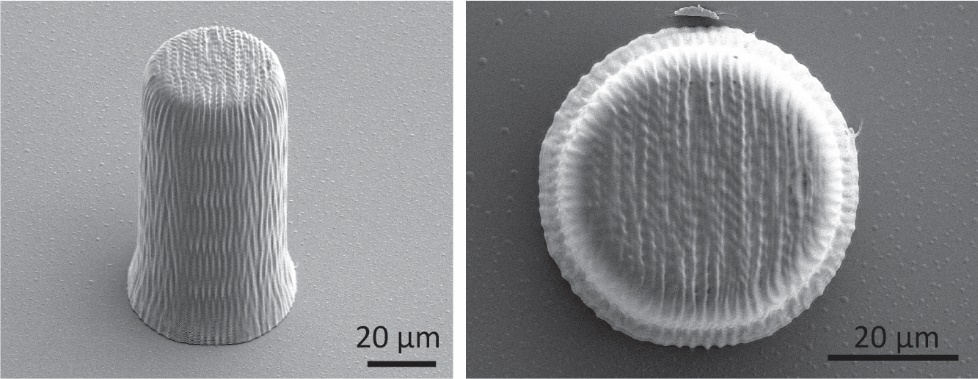
**

**Supplementary Figure 2.** Tilted (left) and top (right) SEM image of the 2PP printed micropillars. Tilt angle = 30°. Scalebar = 20 μm.


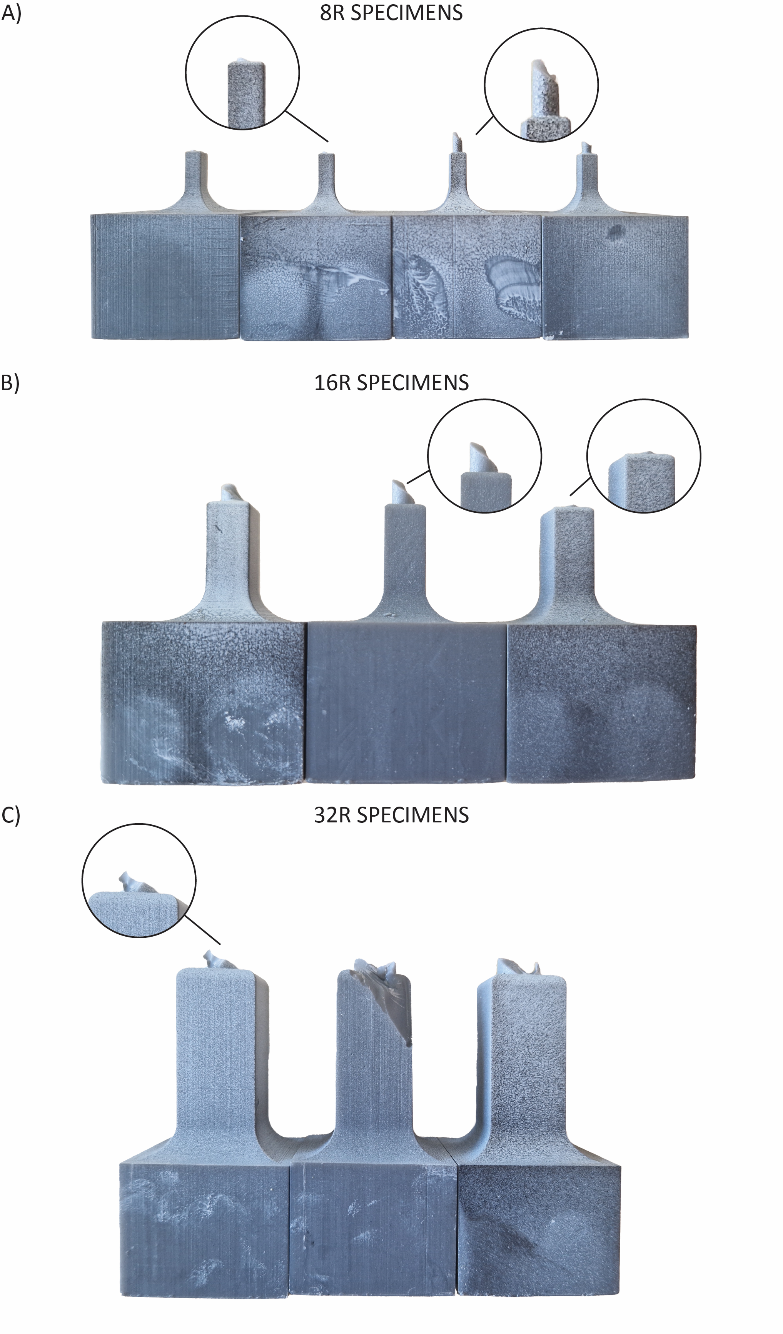


**Supplementary Figure 3.** The optical images depicting the fracture angles of all solid Grey resin torsion specimen types (i.e., 8R, 16R, 32R).

**
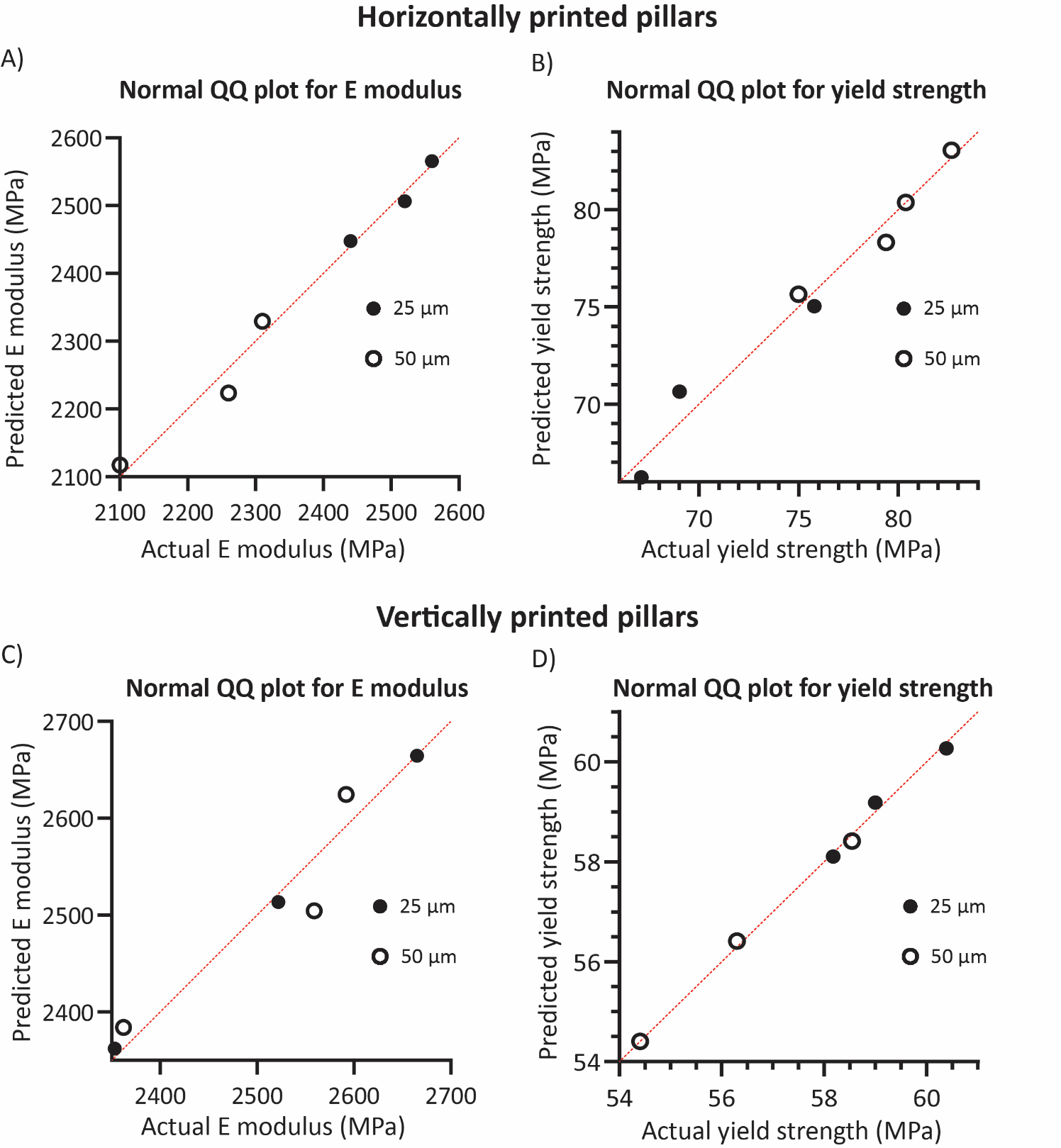
**

**Supplementary Figure 4.** The QQ normality plots for A) the elastic modulus and B) the yield strength of horizontally printed Grey resin pillars with 25 μm and 50 μm layer thickness, and for C) the elastic modulus and D) the yield strength of vertically printed Grey resin pillars with 25 μm and 50 μm layer thickness.


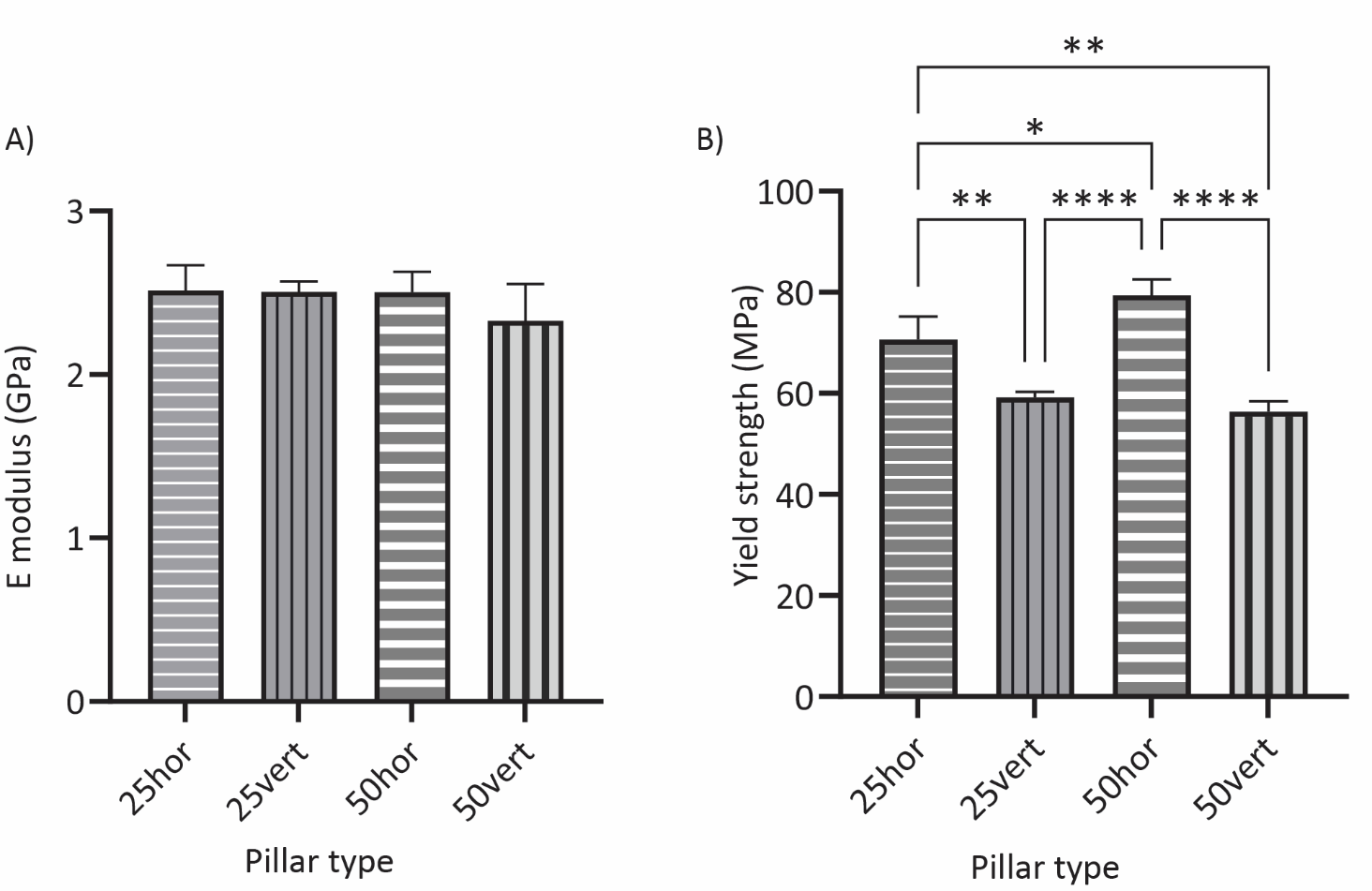


**Supplementary Figure 5.** ANOVA results for A) the Young’s modulus and B) the yield strength of horizontally or vertically printed Grey resin pillars with layer thicknesses of 25 μm and 50 μm. Significance levels are denoted as follows: * for p < 0.05, ** for p < 0.01, *** for p < 0.001, and **** for p < 0.0001.

**Supplementary Table 1.** Fracture angle measurement of the Grey resin torsion specimens in degrees (NM: non-measurable or flat, -: no sample)

| Specimen | 8R | 16R | 32R |
| --- | --- | --- | --- |
| 1 | NM | 37° | 33° |
| 2 | NM | 36° | NM |
| 3 | 44° | NM | 31° |
| 4 | 41° | - | - |

**Supplementary Table 2.** Normality test results from the data exploration step of the compression data of vertically and horizontally printed Grey resin pillars in two different layer thicknesses (i.e., 25 μm and 50 μm). Shapiro-Wilk p-value < 0.05 confirms non-normal data distribution, justifying non-parametric analysis.

| Shapiro-Wilk test for **horizontally** printed pillars | **p-value** | | **Result** |
| --- | --- | --- | --- |
|  | 25 μm | 50 μm | Passed normality? |
| E modulus | 0.6369 | 0.4391 | yes |
| Yield strength | 0.4037 | 0.7335 | yes |
| Shapiro-Wilk test for **vertically** printed pillars | **p-value** | | **Result** |
|  | 25 μm | 50 μm | Passed normality? |
| E modulus | 0.9082 | 0.2541 | yes |
| Yield strength | 0.7156 | 0.9050 | yes |
